# A Lithium–Sulfur Battery Using Binder-Free Graphene-Coated Aluminum Current Collector

**DOI:** 10.1021/acs.energyfuels.2c02086

**Published:** 2022-07-28

**Authors:** Wolfgang Brehm, Vittorio Marangon, Jaya Panda, Sanjay B. Thorat, Antonio Esaú del Rio Castillo, Francesco Bonaccorso, Vittorio Pellegrini, Jusef Hassoun

**Affiliations:** † BeDimensional S.p.A., Lungotorrente Secca 30r, 16163 Genoa, Italy; ‡ Department of Chemical and Pharmaceutical Sciences, University of Ferrara, Via Fossato di Mortara 17, 44121 Ferrara, Italy; § Istituto Italiano di Tecnologia, Via Morego 30, 16163 Genoa, Italy

## Abstract

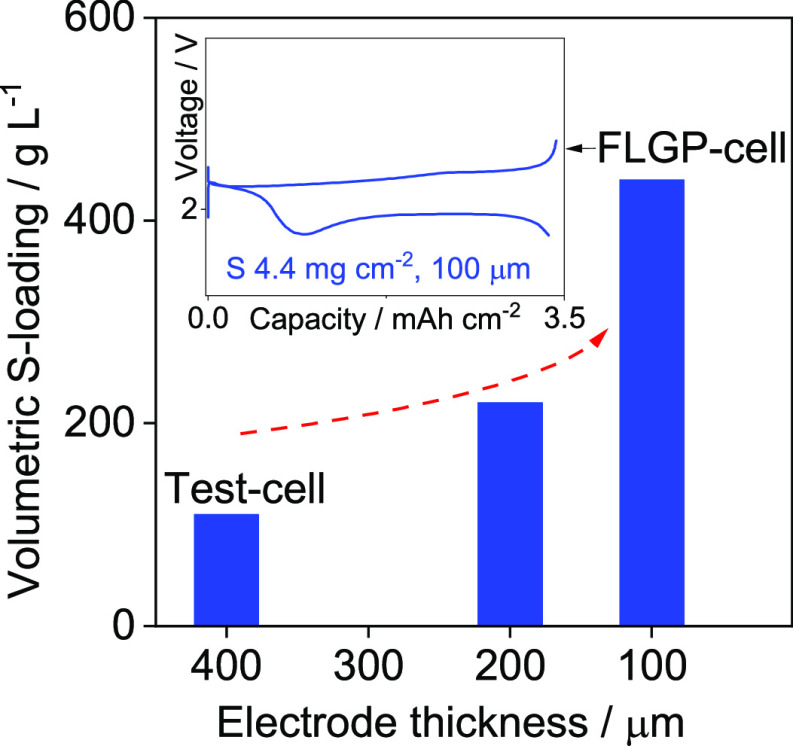

Lithium–sulfur battery of practical interest requires thin-layer support to achieve acceptable volumetric energy density. However, the typical aluminum current collector of Li-ion battery cannot be efficiently used in the Li/S system due to the insulating nature of sulfur and a reaction mechanism involving electrodeposition of dissolved polysulfides. We study the electrochemical behavior of a Li/S battery using a carbon-coated Al current collector in which the low thickness, the high electronic conductivity, and, at the same time, the host ability for the reaction products are allowed by a binder-free few-layer graphene (FLG) substrate. The FLG enables a sulfur electrode having a thickness below 100 μm, fast kinetics, low impedance, and an initial capacity of 1000 mAh g_S_^–1^ with over 70% retention after 300 cycles. The Li/S cell using FLG shows volumetric and gravimetric energy densities of 300 Wh L^–1^ and 500 Wh kg^–1^, respectively, which are values well competing with commercially available Li-ion batteries.

## Introduction

The increase of LIB energy density is nowadays a widely discussed topic in view of their extended application to electric and hybrid vehicles.^1,2^ Moreover, various concerns including toxicity, scarce accessibility, and high cost of transition metals such as Co and Ni may actually hinder the large-scale application of LIBs.^3^ Alternative chemistries, such as those based on alkaline metal conversion or alloying, can lead to higher energy density compared to the lithium (de)intercalation one.^4−6^ Among them, the lithium–sulfur (Li/S) conversion process appears as one of the most promising candidates to achieve lithium battery with enhanced performances compared to the state-of-art. In fact, the sulfur electrode can deliver in a lithium cell a theoretical capacity and energy density as high as 1675 mAh g^–1^ and 2600 Wh kg^–1^,^7,8^ respectively, instead of 280 mAh g^–1^ and 1036 Wh kg^–1^, typical of the commercial layered electrodes, such as LiNi_0.33_Mn_0.33_Co_0.33_O_2_ (NMC).^9^ In addition*,* sulfur holds various bonuses including the large abundance on the earth’s crust, low price, and environmental compatibility.^10^ Sulfur (S_8_) can reversibly operate in a lithium cell through a multi-step electrochemical process, leading to the formation of various polysulfide intermediates, which can be highly soluble (Li_2_S_*x*_, 6 ≤ *x* ≤ 8) or almost insoluble (Li_2_S_2_ and Li_2_S) into the electrolyte media according to the overall reaction: S_8_ + 16Li^+^ + 16e^–^ ⇄ 8Li_2_S.^7,11^ Unfortunately, soluble polysulfides can migrate and directly react with the lithium anode or shuttle between the anode and cathode throughout a continuous process without any charge accumulation.^12,13^ This leads to an efficiency decrease, active material loss, or even to short circuits and cell failure, while insoluble polysulfides can precipitate into the cell and cause resistance increase and capacity fading.^12,13^ It is worth mentioning that the low electronic and ionic conductivity of elemental sulfur triggered its use as composite mainly with carbons,^14−17^ metals,^18−21^ metal oxides,^22−25^ and conductive polymers.^26,27^ Furthermore, the characteristic electrochemical process involving the electro-deposition/dissolution of soluble species at the cathode side focused the attention on the nature of the current collector.^28−30^ Aluminum is typically used as the cathode support in lithium batteries for either insertion or sulfur-based electrodes due to its relevant oxidative stability, promoted by the presence of an Al_2_O_3_ nanometric passivating layer which remarkably protects the metal surface from further reactions and enhances the safety content of the system.^4,31^ However, flat and thin metal supports (*e.g*., bare Al current collector) may lead to poor performances due to high overall impedance of the cell and modest ability in allowing the complex multi-step reaction pathway, while thicker porous supports (*e.g*., gas diffusion layer, GDL) can enhance the cell response, reduce the impedance, and actually boost the kinetics of the Li/S process.^32−34^ In fact, the rough microporous surface of the GDL (micropore area of 0.3 m^2^ g^–1^ with pore volume of 0.04 cm^3^ g^–1^)^33^ can host the active material, allow a continuous contact of sulfur with the current collector, and facilitate the electrochemical reaction of dissolved intermediates.^32^ As a result, GDL shows a higher specific capacity compared to bare Al (1060 vs 770 mAh g^–1^ at C/5 using a graphene-based sulfur composite),^33^ which is typically used as support in LIBs. Despite the fact that GDL can enhance the Li/S cell performances, it may pose concerns linked with the relevant reduction of the volumetric energy density due to the higher thickness compared to bare Al at the cathode side (*i.e.*, 450 vs 18 μm). Carbon coatings on thin metallic substrates can actually reduce the thickness, while holding acceptable gravimetric capacity,^30^ and may involve the use of a polymeric binder.^35,36^ However, excessively porous carbon blends with binder can increase again the thickness, thus vanishing the advantages of the metal support in terms of volumetric energy density. In this view, graphene-based materials may actually allow the thinnest configuration of a carbon-coated metal support and hold, at the same time, a suitable Li/S process due to their characteristic morphology, mechanical stability, and enhanced electronic conductivity.^37^ Herein, we exploit a binder-free few layer graphene (FLG) alcoholic dispersion to design/produce a thin carbon-coated Al support for application in a Li/S cell with excellent characteristics in terms of stability, efficiency, and delivered capacity. The designed battery is expected to have a volumetric energy (*i.e*., 300 Wh L^–1^) comparable to that of the high-performance LIBs but with a superior gravimetric energy density value (*i.e*., 500 Wh kg^–1^). The obtained performances can boost the large-scale diffusion of such energy storage technology.

## Experimental Section

### Preparation of Binder-Free FLG-Coated Al Current Collector

The FLG-coated current collectors were prepared using a few-layer graphene paste (FLGP) obtained by wet-jet mill (WJM) process as reported elsewhere.^38^ Accordingly, FLGP was prepared by dispersing FLG in distilled water with a concentration of 130 g L^–1^ and 1 wt % of sodium deoxycholate (Sigma-Aldrich) as an anionic surfactant to stabilize the FLG flakes.^39,40^ The paste was subsequently diluted using 2-propanol, with two different FLGPs to 2-propanol weight ratios for comparison, *i.e*., 60:40 and 50:50 wt %. The FLGP was then coated on Al foil by means of a doctor blade using a height setting of 200 μm. Subsequently, the coating was dried on a hotplate for 2 h at 75 °C and manually roll-pressed afterward.Table 1 reports the acronyms and characteristics of the obtained FLG-coated Al current collectors in terms of starting composition, final thickness, and weight compared with the bare Al.

**Table 1:** Characteristics of the Al_FLGP Current Collectors in Comparison to Bare Aluminum

current collector	FLGP: isopropanol [% wt]	weight [ mg cm^–2^]	thickness [μm]
bare Al		4.06	18
Al_FLGP_50	50:50 wt %.	4.95	40
Al_FLGP_60	60:40 wt %	5.39	40

### Sulfur Electrode Preparation

Sulfur-Super P carbon (S-SPC) composite was prepared by a mixing-melting process (MP) reported elsewhere.^32^ Accordingly, sulfur (S_8_ ≥ 95%, Riedel-de Haën) and carbon black (Super P C65, TIMCAL) were mixed in the weight ratio of 70:30 with a mortar. The mixture was heated up to 125 °C in a silicon oil bath under magnetic stirring for ∼1 h until complete sulfur melting and subsequent homogenization. The material was cooled down to room temperature and ground using a mortar. The electrode slurry was prepared by mixing the S-SPC composite together with carbon black (Super P C65, TIMCAL) and a polymer binder (PVDF 6020, Solef ) in *N*-methyl-2-pyrrolidone (NMP, Sigma Aldrich), using an 80:10:10 weight ratio. The slurry was cast on the above-prepared FLG-coated Al current collectors and, for comparison, on bare Al using a doctor blade with a height of 250 μm. The electrode foils were subsequently dried at 50 °C for 3 h under ambient conditions using a hotplate. Finally, the electrodes were cut into 14 mm-diameter disks (geometric area of 1.54 cm^2^) and dried under vacuum at 35 °C overnight to remove traces of water and solvent.

### Li/S Cell Assembly and Electrochemical Tests

CR2032 Li/S coin-cells were achieved using Li metal chips (MTI co.) cut with a diameter of 14 mm as the counter/reference electrode and the above-described sulfur working electrode, in an Argon-filled glovebox (MBraun) with H_2_O and O_2_ contents below 1 ppm. The electrolyte was formed by dissolving 1 mol of lithium bis(trifluoromethanesulfonyl)imide (LiN(SO_2_)_2_(CF_3_)_2_, LiTFSI, 99.95% trace metals basis, Sigma-Aldrich) and 1 mol lithium nitrate (LiNO_3_, 99.99% trace metals basis, Sigma-Aldrich) in 1 kg of 1,3-dioxolane/1,2-dimethoxyethane DOL:DME 1:1 w:w mixture (both Sigma Aldrich, 1:1, dried with 3 Å rods, size 1/16 in., Honeywell Fluka, molecular sieves). An electrolyte to sulfur (E/S) ratio of 15 μL mg_S_^–1^ was set using a Celgard 2400 as the separator. The sulfur loading used for material characterization ranged from 1 to 2 mg cm^–2^. Cyclic voltammetry (CV) measurements were conducted with a scan rate of 0.1 mV s^–1^ in a voltage window of 1.8–2.8 V vs Li^+^/Li, while electrochemical impedance spectroscopy (EIS) measurements were performed in a frequency range of 500 kHz to 0.1 Hz with an amplitude of 10 mV, both using a VersaSTAT MC Princeton Applied Research (PAR–AMETEK) analyzer. Galvanostatic cycling was performed by applying a current rate of C/5 (1C = 1675 mA g_S_^–1^) within 1.9–2.8 V using a MACCOR series 4000 battery cycler. An additional cell using FLG-coated Al with sulfur loading increased up to ∼4.4 mg cm^–2^ and an E/S ratio limited to 10 μL mg_S_^–1^ was assembled and tested to further study the practical achievements of the new support.

### Materials Characterization

High-resolution imaging was performed with a JEOL JEM-0100 transmission electron microscope (TEM), operated with an acceleration voltage of 100 kV. The FLG powder was dispersed in NMP with a 1:50 ratio before drop-casting into an ultrathin C-film on holey carbon 400 mesh Cu grids from Ted Pella Inc. Thickness analysis of FLG samples was carried out by utilizing a Park NX10 atomic force microscope (AFM) in a non-contact mode. A silicon probe of frequency of 300 ± 100 kHz and a spring constant of 26 N m^–1^ were used. The FLG dispersion was diluted in 2-propanol in a volumetric ratio of 1/20 and drop-casted into mica wafers. The wafers were subsequently dried at 100 °C overnight. The thickness profiles of more than 100 flakes were considered for calculation by measuring several images at different regions of the samples. Thermogravimetric analysis (TGA) measurements were performed under a N_2_ atmosphere using a heating rate of 5 °C min^–1^ in the 25–800 °C temperature ranges through a Mettler–Toledo TGA 2 instrument. Raman measurements were carried out by a Renishaw InVia micro-Raman spectrometer with a 100× objective (numerical aperture of 0.85). An excitation wavelength of the 514.5 nm line of a diode laser was used with an incident power of less than 1 mW striking on the sample to avoid heating effects. The scattered light was detected in a back-scattering geometry dispersed by a grating of 24,000 grooves/mm. Scanning electron microscopy (SEM) was conducted with a JEOL JSM-6490LA SEM analytical (low-vacuum) instrument with a thermionic electron gun equipped with a tungsten source.

## Results and Discussion

**Figure 1:**
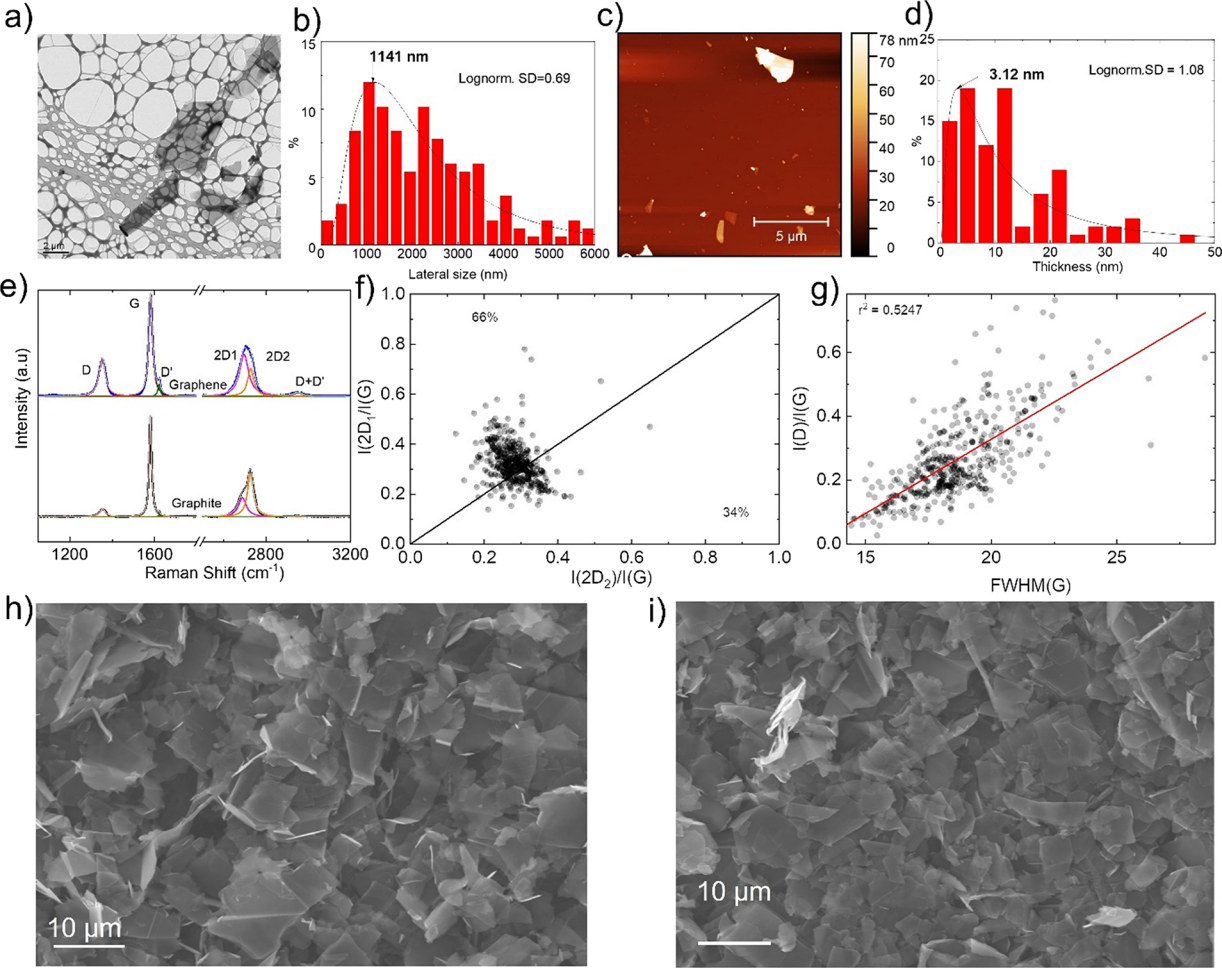
(a) Transmission electron microscopy (TEM) image, (b) statistical lateral size distribution with Lorentzian fit, (c) atomic force microscopy (AFM) image, and (d) statistical thickness distribution of the FLG precursor. (e) Raman spectrum of the FLG precursor including graphite reference with (f) corresponding *I*(2*D*_1_)/*I*(*G*) vs *I*(2*D*_2_)/*I*(*G*) and (g) statistical Raman analysis of *I*(*D*)/*I*(*G*) vs FWHM(*G*). SEM images of (h) Al_FLGP_50 and (i) Al_FLGP_60 after coating and pressing. See Table 1 for the definition of the samples’ acronyms.

The as-produced graphene-based materials are investigated by TEM, AFM, and SEM, see Figure 1. The flat morphology of the single flake is clearly evidenced by the TEM image of Figure 1a, thus in full agreement with previous reports.^14,41−44^ Statistical analysis of the lateral size distribution, obtained using a Lorentzian fit, shows a maximum peak at ∼1141 nm (Figure 1b). Furthermore, the AFM response allows the determination of the thickness of the flakes, see Figure 1c. The statistical analysis reveals a flake thickness distribution with a maximum centered at ∼3.12 nm with a lognormal distribution of 1.08 (Figure 1d).^14,45^ The Raman spectrum of the as-produced graphene-based materials is reported in Figure 1e, together with the one of graphite plotted as reference, evidencing the typical three main peaks. These are, the *G* peak at ∼1580 cm^–1^ corresponding to the *E*_2g_ phonon at the Brillouin zone center,^46^ the *D* peak at 1350 cm^–1^ correlated with the symmetry breaking of the sp^2^ carbon rings, and the peak at around 2700 cm^–1^ due to the second-order of the D peak (2*D*).^14,47^ The Raman spectrum of the as-produced graphene-based materials shows the typical features expected for the single-layer graphene (SLG) and few/multi-layer graphene flakes.^45^ In fact, if compared with the Raman spectrum of graphite, the G and 2D Raman peaks change in shape, position, and relative intensity.^38^ This reflects the evolution of the electronic structure with the number of graphene layers.^38^ In this respect, the 2*D_1_* to 2*D_2_* intensity ratio (Figure 1f) allows the estimation of the flake thickness.^38^ The graphite reference shows an intensity of the *I*(2*D*_2_) peak which is about double of the intensity of the *I*(2*D*_1_) peak, while the intensity typically decreases and modifies when the thickness drops until the 2*D* band can be fitted by a single Lorentzian plot as the flakes are electronically decoupled.^14,47^ Hence, the line *I*(2*D*_1_)/I(*G*) = *I*(2*D*_2_)/*I*(*G*) indicates a staking of more than five layers for the FLG,^14,48^ in which the values above the line *I*(2*D*_1_)/*I*(*G*) < *I*(2*D*_2_)/I(*G*) belong to FLG (66%) and the points below I(2*D*_1_)/I(*G*) > I(2*D*_2_)/I(*G*) to graphite (34%). Furthermore, the absence of a linear correlation between FWHM(*G*) and the normalized *I*(*D*)/*I*(*G*) band in the statistical Raman analysis (Figure 1g) suggests the presence of sub-micrometric flakes as well as the absence of structural defects.^14,47^ In addition, the SEM imaging performed at the surface of the Al_FLGP_50 (Figure 1h) and Al_FLGP_60 (Figure 1i) current collectors shows a more relevant stacking of the FLG in the support compared to the above discussed Raman of the graphene precursor, and a porous morphology, which is influenced by the amount of isopropanol used for the preparation of the sample (see Experimental Section for preparation details and Table 1 for the definition of the acronyms).

Despite the notable packing of the FLG, the Al_FLGP_50 and Al_FLGP_60 show a reasonably small thickness (40 μm) and low carbon loading (*i.e*., of ∼1 mg_FLG_ cm^–2^) due to the relevant water and isopropanol evaporation during the preparation process (see corresponding TGA and differential thermal analysis -DTG- in Figure S1 in the Supporting Information) and roll pressing process. Hence, the Al_FLGP supports reveal comparable characteristics to those of the bare Al used in LIBs (see Table 1), in addition to a notable mechanical stability and a modest porosity (see corresponding BET in Figure S2 in the Supporting Information). These can be considered optimal features, which are expected to enhance the performances of the Li/S cell and, at the same time, hold a suitable volumetric energy density as reported hereafter.

**Figure 2:**
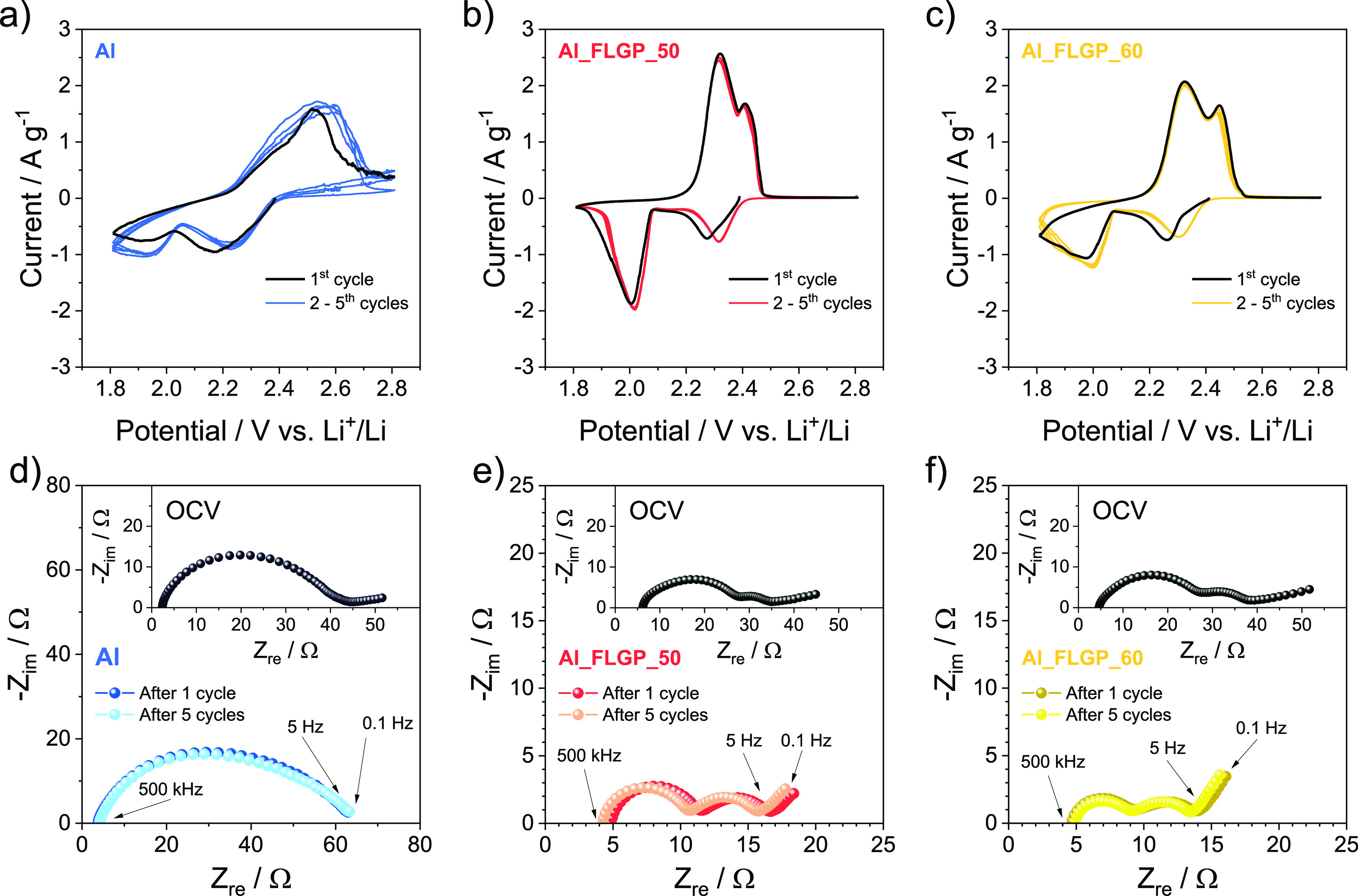
Cyclic voltammetry (CV) measurements with currents normalized to the weight of the sulfur in the electrodes using (a) bare Al, (b) Al_FLGP_50, and (c) Al_FLGP_60 in a potential window of 1.8–2.8 V vs Li^+^/Li at a scan rate of 0.1 mV s^–1^. Nyquist plots of the electrochemical impedance spectroscopy (EIS) performed at the OCV, and upon the first and fifth CV cycle of sulfur electrodes coated on (d) bare Al, (e) Al_FLGP_50, and (f) Al_FLGP_60 in a frequency range from 500 kHz to 0.1 Hz using a signal amplitude of 10 mV. Electrolyte: DOL:DME (1:1 w:w), LiTFSI (1 mol kg^–1^), LiNO_3_ (1 mol kg^–1^). Room temperature (25 °C). See Table 1 for samples’ acronyms.

The role of FLG on the Li/S reaction kinetics is studied by coupling cyclic voltammetry and electrochemical impedance spectroscopy as reported in Figure 2. The voltammograms (Figure 2a–c) reveal peaks during the cathodic scan at potentials of ∼2.0 and 2.3 V vs Li^+^/Li likely ascribed to the reduction of the S_8_ rings to long chain polysulfides (possibly S_8_ + 2Li+ + 2e- ⇄ Li_2_S_8_) and short chain polysulfides (Li_2_S_8_ + 14Li+ + 14e- ⇄ 8Li_2_S), while the oxidation back of polysulfides to sulfur and lithium takes place during the reverse anodic scan at potentials higher than 2.2 V vs Li^+^/Li.^49^ However, according to the previous literature, the two discharge processes at ∼2.0 and 2.3 V vs Li^+^/Li hardly correspond to single oxidation states of the Li_2_S_*x*_ polysulfides, since the conversion from Li and S to Li_2_S actually occurs through the formation of various intermediate anions and free radical species into a complex equilibrium.^50^ The shape of the first cycle slightly differs from the subsequent ones for all samples, likely due to a structural reorganization occurring during the conversion process at the cathode side and to the possible SEI formation at the electrodes surface.^18^ The CV curves reveal a less defined profile and a higher polarization for the cell using a bare Al current collector compared to Al_FLGP_50 and Al_FLGP_60. After the first CV scan, the cell using Al shows a depressed reduction wave occurring at potentials of ∼1.94 and ∼ 2.20 V vs Li^+^/Li and a single broad oxidation peak at ∼2.52 V vs Li^+^/Li (Figure 2a). Instead, the cell based on Al_FLGP_50 support is characterized by narrow reduction peaks at ∼2.01 and ∼2.32 V vs Li^+^/Li with a reverse oxidation according to a defined double peak at ∼2.32 and ∼2.41 V vs Li^+^/Li (Figure 2b). Concerning the cell using Al_FLGP_60, it shows a similar but less intense response compared to the Al_FLGP_50 one, with reduction peaks at ∼2.00 and ∼2.31 V vs Li^+^/Li and oxidation at ∼2.32 and ∼2.45 V vs Li^+^/Li (Figure 2c). The above CV responses clearly indicate that the FLG coating on Al is contributing to enhance the conversion kinetics in the Li/S cell, most likely due to the favorable features of the FLG in terms of morphology, electronic conductivity, and hosting ability to the reaction products*.*^51^ Furthermore, the cell using Al_FLGP_50 shows more intense CV signals compared to the one based on Al_FLGP_60 despite the similar morphology observed in Figure 1, which may be ascribed to slightly different surfaces, as indeed observed by BET data reported in Figure S2 (Supporting Information). The reasons for the Li/S cell kinetic improvement achieved by FLG coating on Al can be in part rationalized by the EIS Nyquist plots performed upon CV in the 500 kHz–0.1 Hz frequency range (Figure 2d–f), in which the charge transfer and SEI are represented by semicircles positioned in a middle-high frequency range, while possible Li^+^-ion diffusion is typically shown as a linear progression in the low-frequency region.^52,53^ The corresponding resistance values are calculated by nonlinear least squares (NLLS) analysis and listed in Table 2. The data at the open circuit voltage (OCV) condition reveal an overall interphase resistance (*R* = *R*_1_ + *R*_2_ in Table 2) of 49.1 Ω for the Li/S cell using a bare Al current collector (Figure 2d); instead, values of 29.1 Ω and 34.0 Ω are observed for the cells using Al_FLGP_50 (Figure 2e) and Al_FLGP_60 (Figure 2f), respectively. Notably, the cell based on bare Al reveals an increase of R to ∼61 Ω after five CV cycles, which is likely ascribed to the formation of an unfavorable electrode/electrolyte interphase upon polysulfide dissolution.^33^ In contrast, the Li/S cells with Al_FLGP_50 and Al_FLGP_60 show a resistance decrease to ∼11 and ∼8 Ω, respectively, after the same number of voltammetry cycles, which is in line with the smaller polarization observed by the ongoing of the CV test (see Figure 2b,c). The resistance decrease and consequent cell improvement upon cycles may be ascribed to activation processes described in previous reports,^18,20^ leading to a favorable electrode/electrolyte interphase and enhanced charge transfer kinetics. These processes are promoted by the homogeneous distribution of sulfur into the carbon framework upon repeated sulfur electrodeposition and polysulfide dissolution. These advantageous features suggest the FLG coating on the Al current collector as an adequate strategy to achieve enhanced Li/S cells using a thin sulfur cathode.

**Table 2:** NLLS Analyses Performed on the Nyquist Plots (Figure 2d–f) by Using a Boukamp Tool^52,53^ (See Table 1 for Samples’ Acronyms)

material	cell condition	equivalent circuit	*R*_1_ [Ω]	*R*_2_ [Ω]	*R*_1_ + *R*_2_	χ^2^
Al	OCV	R_e_(R_1_Q_1_)(R_2_Q_2_)	35.2 ± 2.3	13.9 ± 4.6	49.1 ± 6.9	6 × 10^–4^
	1 CV cycle	R_e_(R_1_Q_1_)(R_2_Q_2_)	31.2 ± 8.5	29.5 ± 8.7	60.7 ± 17.2	9 × 10^–5^
	5 CV cycles	R_e_(R_1_Q_1_)(R_2_Q_2_)	33.0 ± 4.7	28.0 ± 5.0	61 ± 9.7	5 × 10^–5^
Al_FLGP_50	OCV	R_e_(R_1_Q_1_)(R_2_Q_2_)Q_w_	22.9 ± 0.3	6.2 ± 0.4	29.1 ± 0.7	8 × 10^–5^
	1 CV cycle	R_e_(R_1_Q_1_)(R_2_Q_2_)Q_w_	5.9 ± 0.2	6.0 ± 0.6	11.9 ± 0.8	5 × 10^–4^
	5 CV cycles	R_e_(R_1_Q_1_)(R_2_Q_2_)Q_w_	5.9 ± 0.2	5.5 ± 0.4	11.4 ± 0.6	3 × 10^–4^
Al_FLGP_60	OCV	R_e_(R_1_Q_1_)(R_2_Q_2_)Q_w_	22.4 ± 0.7	11.6 ± 1.2	34.0 ± 1.9	4 × 10^–4^
	1 CV cycle	R_e_(R_1_Q_1_)(R_2_Q_2_)Q_w_	3.6 ± 0.2	5.9 ± 0.5	9.5 ± 0.7	5 × 10^–4^
	5 CV cycles	R_e_(R_1_Q_1_)(R_2_Q_2_)Q_w_	3.4 ± 0.2	5.2 ± 0.4	7.6 ± 0.6	5 × 10^–4^

**Figure 3:**
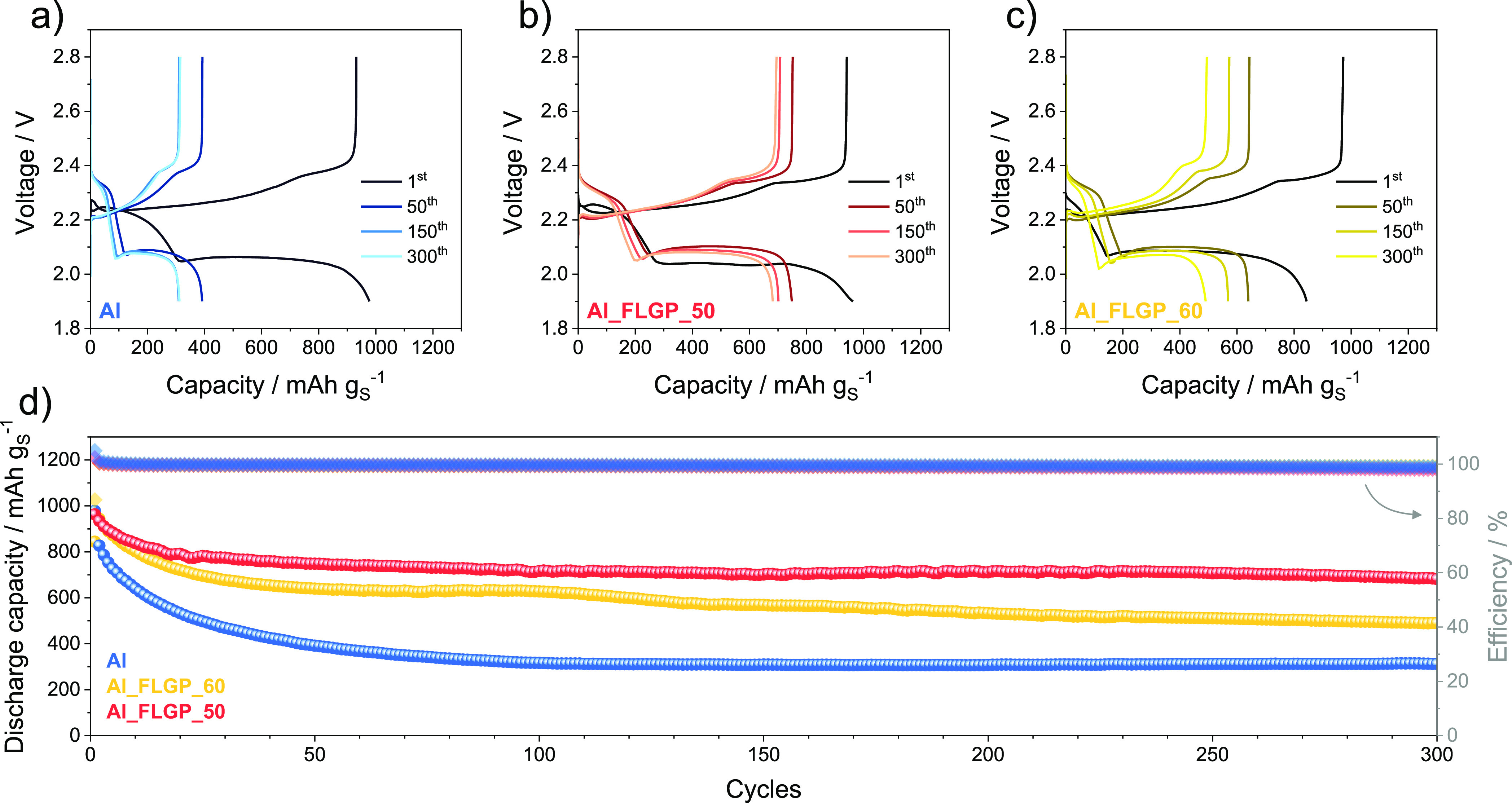
Voltage profiles of Li/S cells cycled at a C/5 rate (1C = 1675 mA g_S_^–1^) using electrodes coated on (a) bare Al, (b) Al_FLGP_50, and (c) Al_FLGP_60. (d) Comparison of the corresponding discharge capacity trends upon cycling (left-side *y*-axis) and Coulombic efficiency (right-side *y*-axis). Electrolyte: DOL:DME (1:1 w:w), LiTFSI (1 mol kg^–1^), LiNO_3_ (1 mol kg^–1^). Voltage window 1.9–2.8 V. Room temperature (25 °C). See Table 1 for samples’ acronyms.

The galvanostatic responses of the Li/S cells using different current collectors are displayed in Figure 3. The voltage profile of the test performed at a C/5 rate (1 C = 1675 mA g_S_^–1^) on a cell with bare Al support (Figure 3a) reveals the typical two-plateau signature of the Li/S battery during the initial stages, reflecting the polarization expected by the corresponding CV curves (compare with Figure 2a).^8^ However, the voltage profile reveals a remarkable fading of the reversible capacity from 980 mAh g_S_^–1^ at the first cycle to values lower than 310 mAh g_S_^–1^ after 300 cycles. On the contrary, the cells based on Al_FLGP_50 and Al_FLGP_60 show lower polarization and higher stability compared with the reference one. In fact, despite the similar capacity of 965 mAh g_S_^–1^ at the first cycle, the cell using Al_FLGP_50 shows at the same C-rate a capacity approaching 700 mAh g_S_^–1^ at the 300th cycle (Figure 3b), while the one using Al_FLGP_60 reveals a lower capacity of 843 mAh g_S_^–1^ at the first cycle, decreasing to 490 mAh g_S_^–1^ over 300 cycles (Figure 3c). Figure 3d, showing the comparison of the discharge capacity with the efficiency of the three cells, clearly evidences the stabilization effect of the Li/S battery achieved by the FLG coating of Al. In fact, bare Al leads to an average capacity loss of 2.23 mAh g_S_^–1^ per cycle, while Al_FLGP_50 and Al_FLGP_60 limit the decay to 0.91 mAh g_S_^–1^ and 1.18 mAh g_S_^–1^ per cycle, respectively. It is worth mentioning that the capacity values achieved herein are lower than those achieved by the carbon current collectors typically used with the aim of active material characterization rather than practical cell application, such as GDL.^32^ The latter is thicker compared to Al (*i.e*., 450 μm vs 18 μm) and much more porous, thus allowing the S-SPC composite used herein to exceed 1000 mAh g_S_^–1^.^32^ However, the excessive thickness of the above support may limit the application of the Li/S cell due to a resulting low volumetric energy density despite the high gravimetric value. Instead, the cells using FLG-coated Al reveal very promising values of the gravimetric capacity with stability and thickness values that may lead to competing volumetric energy density.

**Figure 4:**
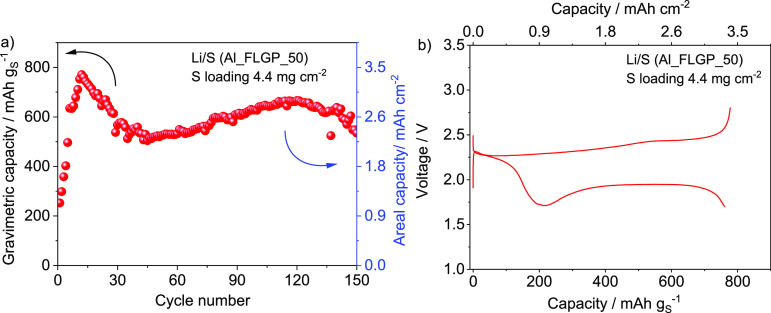
(a) Gravimetric (mAh g^–1^, left-side *y*-axis) and areal (mAh cm^–2^, right-side *y*-axis) capacities of the Li/S cell cycled at a C/5 rate (1C = 1675 mA g_S_^–1^) using a S-Al_FLGP_50 cathode with an areal sulfur loading increased up to ∼4.4 mg cm^–2^ (electrode geometric area of 1.54 cm^2^) and (b) voltage profile during a related cycle with the maximum capacity. Electrolyte: DOL:DME (1:1 w:w), LiTFSI (1 mol kg^–1^), LiNO_3_ (1 mol kg^–1^). Voltage window 1.7–2.8 V. Room temperature (25 °C). See Table 1 for samples’ acronyms.

The Li/S cell based on the most performing S-Al_FLGP_50 cathode has been selected for further improvement in terms of areal sulfur content by increasing the loading to ∼4.4 mg cm^–2^ (electrode geometric area of 1.54 cm^2^) and is reported in Figure 4. The cell has an E/S ratio limited to 10 μL mg_S_^–1^, S to graphene-coating ratio increased up to ∼5:1, and S to overall-support ratio of ∼1:1, according to the data reported in Table 1 for the Al_FLGP_50 support. The Li/S cell described above reveals an initial capacity of ∼250 mAh g_S_^–1^, increasing to ∼800 mAh g_S_^–1^, and subsequently stabilizing to ∼650 mAh g_S_^–1^, except for the occasional decrease of the cell performance (Figure 4a) during the initial cycles due to the high voltage cutoff used herein in discharge to avoid side reductive processes as displayed in Figure S3 (Supporting Information). This can be considered as an activation process, in line with the one described in Figure 2 and in previous reports, and becomes more relevant when the sulfur content in the electrode is increased.^18,20^ The figure shows a maximum areal capacity exceeding 3 mAh cm^–2^ (see corresponding voltage profile in Figure 4b), which is a value that can reflect a relevant improvement of the cell volumetric energy density achieved herein by FLG coating even considering reduction factors linked with the inactive component contributions.^54^ Therefore, the data of our work suggest the use of FLG-coated Al as the preferential support for application in practical and scalable Li/S batteries of high energy density and expected modest economic impact.^8^ In fact, the present Li-ion battery has a predicted cost that can be considerably decreased, and the driving autonomy estimated in EVs using LIBs can be enhanced by using a scaled up and efficient Li/S battery.^55^ In addition, the stability of the cell observed in Figure 3 indicates that the new support can guarantee sufficient cycling life of the battery and competing performance compared to the widely diffused LIBs.

## Conclusions

FLG-coated Al supports exploiting a binder-free configuration have been studied as the current collectors for Li/S batteries of practical interest. TEM, SEM, AFM, Raman, and TGA measurements evidenced the characteristic structure and morphology of the new current collectors. Furthermore, cyclic voltammetry and electrochemical impedance spectroscopy have shown limited overpotentials and interphase resistances of the Li/S cells using the FLG-coated supports compared to bare Al. Noteworthy, galvanostatic cycling revealed a stable trend with a specific capacity ranging from ∼950 to ∼700 mAh g_S_^–1^ over 300 cycles for the best current collector, *i.e*., Al_FLGP_50, at a C/5 rate. The obtained electrochemical performances have been attributed to the beneficial impact of morphological (*i.e*., thin) and electrical (highly conducting) properties of the FLG flakes on the kinetics of the Li/S electrochemical conversion process, as well as to the formation of a suitable electrode/electrolyte interphase in the corresponding cells. The above system revealed a maximum areal capacity exceeding 3 mAh cm^–2^, which can be actually reflected into an improved cell volumetric energy density. Therefore, the results reported herein suggest the FLG-coated Al current collector as the substrate of choice for further development of scalable and high-performance lithium sulfur batteries.

## Supporting Information Available

Thermogravimetric analysis (TGA) and differential thermal analysis (DTG) under N_2_ of the FLGP precursor and FLGP-isopropanol mixtures used for the FLGP-Al (Figure S1); Brunauer, Emmett, and Teller (BET) measurements of FLGP-Al (Figure S2); occasional cell performance decrease during the initial cycles of the Li/S system using Al_FLGP_50 with S loading of 4.4 mg cm^–2^ (Figure S3) (PDF)
