# Supplementary material for: A Lithium–Sulfur Battery Using Binder-Free Graphene-Coated Aluminum Current Collector
Source: Energy Fuels. 2022 Jul 28;36(16):9321–8. doi: 10.1021/acs.energyfuels.2c02086 (PMC9394755; doi:10.1021/acs.energyfuels.2c02086)
Supplement: Supplementary file 1 — ef2c02086_si_001.pdf [file ef2c02086_si_001.pdf]

A Lithium-Sulfur Battery Using Binder-Free Graphene-Coated Aluminum Current Collector

Wolfgang Brehm[a,#], Vittorio Marangon[b,c#], Jaya Panda[a], Sanjay B. Thorat[a], Antonio Esaú del Rio Castillo[a], Francesco Bonaccorso[a,*], Vittorio Pellegrini[a], Jusef Hassoun[b,c*]

[a]*BeDimensional S.p.A., Lungotorrente Secca 30r, 16163 Genoa, Italy*

[b]*Department of Chemical and Pharmaceutical Sciences, University of Ferrara, Via Fossato di Mortara 17, 44121, Ferrara, Italy*

[c]*Istituto Italiano di Tecnologia, Via Morego 30, 16163 Genoa, Italy*

**Correspondence e-mail:*

jusef.hassoun@unife.it; jusef.hassoun@iit.it (J. Hassoun)*; f.bonaccorso@bedimensional.it* (F. Bonaccorso)

[#]the authors W. Brehm and V. Marangon contributed equally to this work

Supporting Information

Number of pages: 4.

Number of figures: 3.

Figure S1 reports the thermogravimetric analysis (TGA) and the corresponding curves of differential thermal analysis (DTG) performed under N_2 atmosphere of the few-layer graphene paste (FLGP) precursor and FLGP-isopropanol mixtures used for the preparation of the FLG-Al coated current collectors.

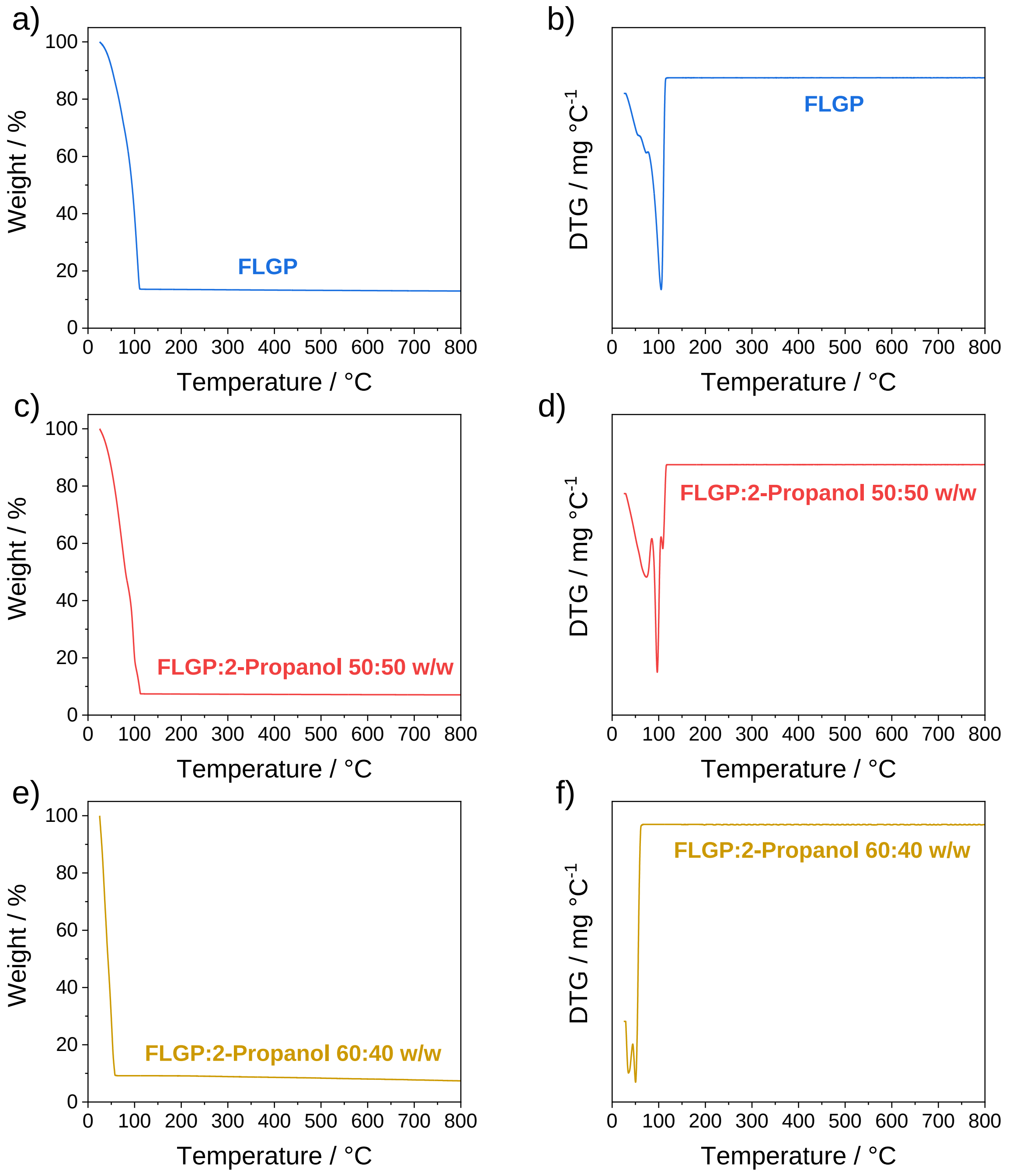

Figure S1. **(a-f)** Weight percent loss of **a)** FLGP, **c)** FLGP_50 **e)** and FLGP_60 mixtures, and corresponding DTG curves in panels **b), d), f)**, respectively. Measurements performed under N_2 using a heating rate of 5 °C min^{-1}. See Table 1 in the manuscript for the definition of the acronyms of the samples.

Figure S2 shows the Brunauer, Emmett and Teller (BET) measurements performed on the FLG-coated Al supports. The measurements show a surface area as low as 0.85 m^2/g for Al_FLGP_50 (Fig. S2a) and of 1.32 m^2/g for Al_FLGP_60 (Fig. S2b) that may allow advantageous characteristic for improving the volumetric energy density of the Li/S cell. See discussion of Figure 1 in the manuscript for further morphological details of the two supports.

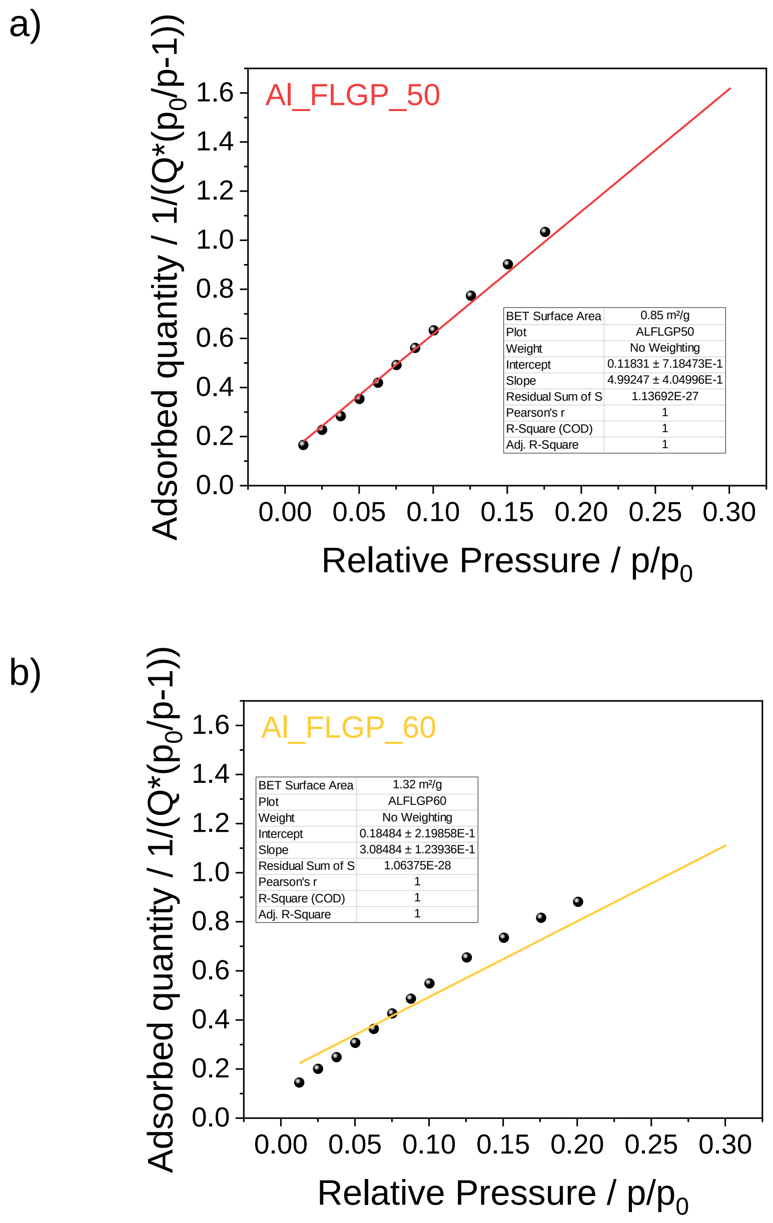

Figure S2. **(a, b)** Brunauer, Emmett and Teller (BET) measurements of **a)** Al_FLGP_50 and **b)** Al_FLGP_60 under nitrogen flow at 90 °C. See experimental section of the manuscript for samples' acronyms.

Figure S3 evidences some of the occasional cell performance decrease detected during the few initial cycles of the Li-S system using the support Al_FLGP_50 with sulfur loading increased up to a value of 4.4 mg cm^{-2} (see Figure 4a in the Manuscript) due to the relatively high discharge voltage cutoff used herein to avoid side reductive processes.

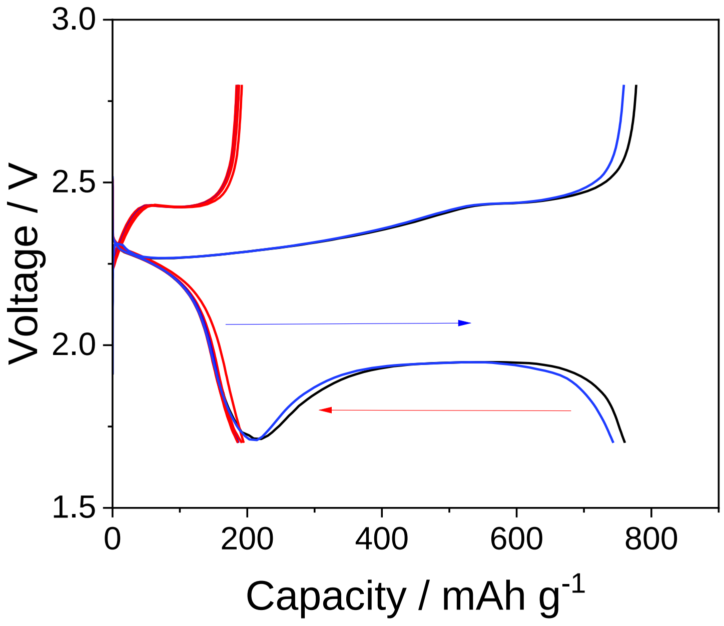

Figure S3. Voltage profiles of the Li/S cell cycled at C/5 rate (1C = 1675 mA g_S^{-1}) using the S-Al_FLGP_50 cathode with an areal sulfur loading increased up to about 4.4 mg cm^{-2} (electrode geometric area of 1.54 cm^2) during the activation process (see Figure 4a in the Manuscript). Electrolyte: DOL:DME (1:1 w:w), LiTFSI (1 mol kg^{-1}), $LiNO_3$ (1 mol kg^{-1}). Voltage window 1.7 – 2.8 V. Room temperature (25°C).
